# Arterioureteral Fistula: Treatment of a Hemorrhagic Shock with Massive Hematuria by Placing a Balloon Catheter

**DOI:** 10.1155/2017/9453618

**Published:** 2017-03-29

**Authors:** Nicolas Merzeau, Hervé Riquet, Ioannis Nicolacopoulos, Abbas Alame, Stéphane Larré

**Affiliations:** ^1^CHU Reims, Hôpital Robert Debré, 51092 Reims, France; ^2^CH Charleville-Mézières, 08011 Charleville-Mézières, France

## Abstract

Arterioureteral fistulas (AUF) are serious diseases with increasing incidence. This case report relates the management of AUF in a patient with a history of abdominal oncological surgery, pelvic radiotherapy, and a double J stent in place. The fistula was discovered during a hemorrhagic shock with massive hematuria. The bleeding was controlled by a balloon catheter which led to endovascular treatment consisting of a covered stent.

## 1. Introduction

Arterioureteral fistulas (AUF) are a rare cause of macroscopic bleeding, reported in about a hundred cases [1]. They can be deadly and reveal questions in regard to how they are managed in emergencies, precise topographic diagnosis, and choice of treatment.

This case concerns the management of secondary AUF, involving the right common iliac artery, in a patient with a history of abdominal oncologic surgery, pelvic radiotherapy, and an indwelling double J right stent. The AUF was discovered due to a macroscopic hematuria complicated by a hemorrhagic shock and was treated with an ureteral balloon catheter and a covered vascular stent.

## 2. Case Report

A 52-year-old female patient consults in 2007 for recurrent hematuria. Her medical history mainly indicates a stenosis of the right ureter in 2004 due to a lymph-node recurrence, located in the aortoiliac intersection, of a rectal adenocarcinoma which was treated by chemoradiotherapy and surgical resection one year earlier. Urine was diverted using a ureteral stent changed regularly.

The hematuria was scarce but persisted despite the establishment of a bladder catheter and irrigation. Suddenly, on the fourth day of hospitalization, a massive hematuria appeared. Physical examination revealed signs of right renal colic and a drop in blood pressure to 70/40 mmHg. A cystoscopy under general anesthesia after hemodynamic stabilization and clot removal was urgently carried out.

It showed a large clot externalized by the right ureteral meatus around the lower end of the ureteral stent. After its removal, pulsatile bleeding was observed by the right ureteral meatus along with a further drop in blood pressure. An arterioureteral fistula was mentioned.

**Figure 1:**
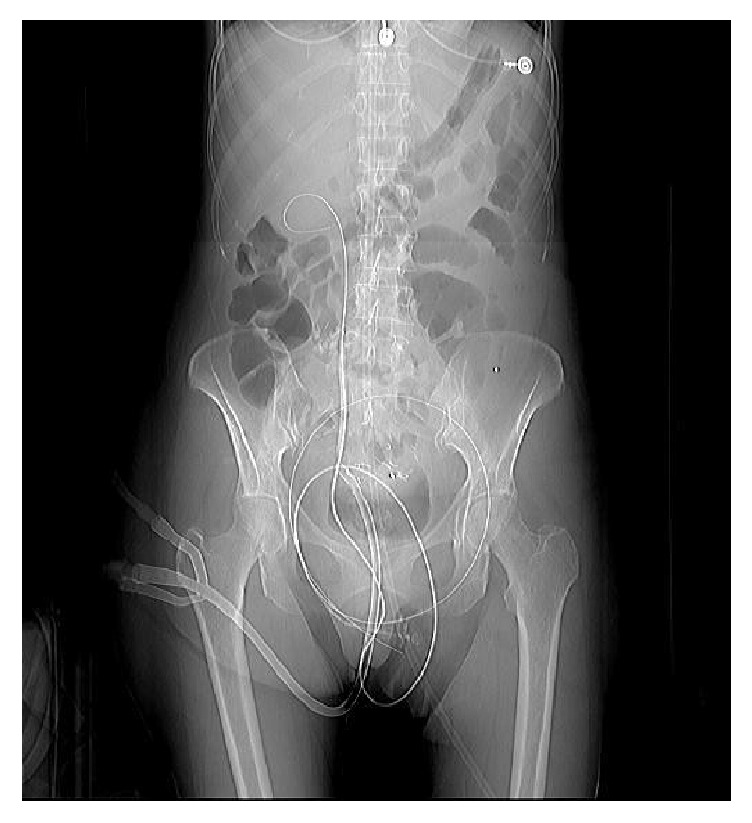
X-ray: probe with endoureteral balloon in place in the right ureter at the level of the fistula with the right primitive iliac artery.

The opacification of the renal pelvis and ureteral cavities showed many pelvic clots but no intravascular path. An ureteral dilatation balloon Ch6 10 cm (mark Bard Uroforce model, reference: 888510) was then positioned at the iliac intersection under fluoroscopic control (Figure 1). After filling the ureteral balloon, bleeding stopped with normalization of the hemodynamic status.

**Figure 2:**
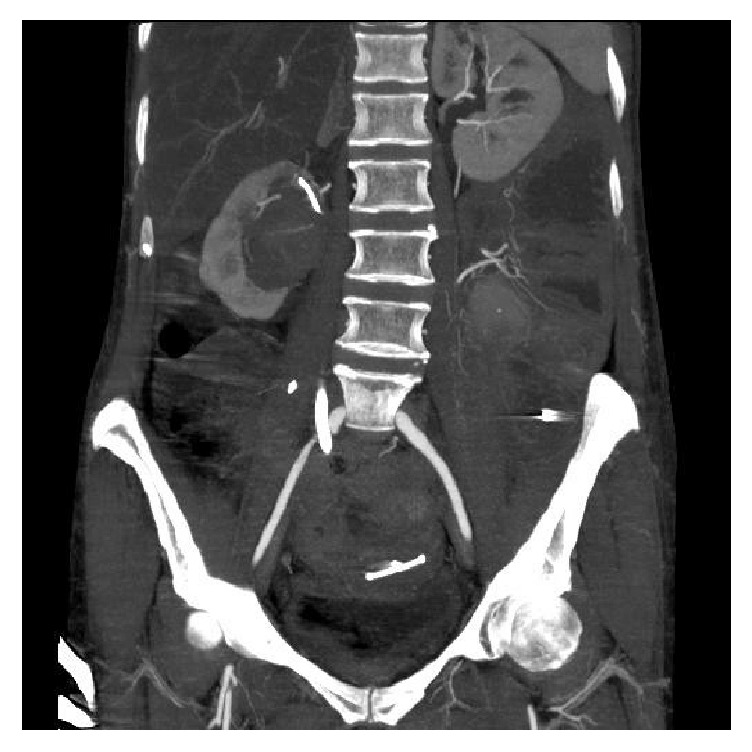
Abdominal-pelvic CT scan frontal cut passing through the right ureter: endoureteral balloon probe in place in the right ureter at the fistula with the right primary iliac artery.

**Figure 3:**
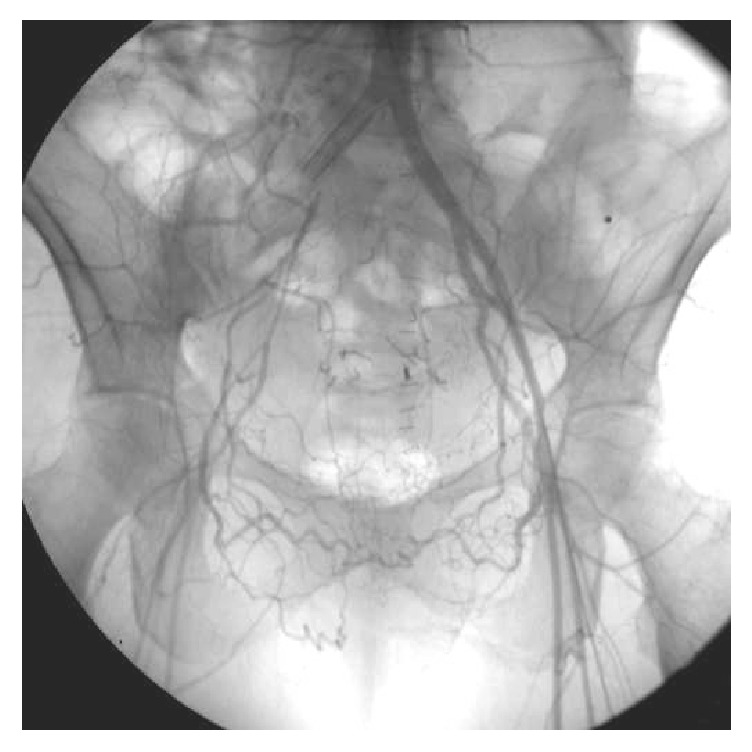
Arteriography of the aortoiliac intersection: endovascular prosthesis covered in place at the level of the right primitive iliac artery, a network of collateral arteries supplying the lower right limbs.

An abdominopelvic CT confirmed the diagnosis of an arterioureteral fistula with the right common iliac artery and the proper positioning of the balloon catheter (Figure 2). A covered stent-graft of the right common iliac artery was then set up about six hours later. A deflated balloon control verified the absence of bleeding at the ureteral fistula. The balloon catheter was replaced with a new ureteric stent after a period of 48 hours of observation with no recurrence of bleeding (Figure 3).

The change was initially favorable but after a year the patient had an inguinal and retroperitoneal abscess associated with an externalization of the stent and thrombosis of the iliac artery. The external and internal iliac arteries were perfused by numerous collaterals. The prosthesis was then removed and the artery bound.

Eight years after treatment of the fistula, the patient showed no vascular sequelae but, urologically, things had evolved towards a gradual destruction of the functional capacity of the right kidney after repeated episodes of pyelonephritis. The ureteral stent had to be removed due to local septic complications and a right nephrectomy was performed in March 2015, with no complications.

## 3. Discussion

The arterioureteral fistulas are rare, but their incidence is steadily increasing due to the proliferation of vascular and urologic practices [2]. Reported risk factors are vascular or abdominal oncologic surgery, ureteral obstruction or prolonged ureteral catheterization, and abdominal-pelvic radiation therapy [2, 3]. Almost all are found in this case report.

Treatment of these fistulas may be achieved with open surgery or by endovascular techniques. Open surgery techniques involve simple arterial ligation or ligation associated with femorofemoral bypass, direct suture, extra-anatomic bypass surgery, or angioplasty by patch [4]. Interventional radiology makes it possible to consider embolization or the use of a covered endovascular stent [4].

Madoff et al., in a literature review, found that the mortality rate in the absence of preoperative diagnosis was nearly 64% but fell to a mere 8% when UAF was identified before surgery [5].

Furthermore, Das et al. in a review of the literature on the AUF found that the most effective modality for imaging is selective arteriography; indeed direct extravasation of contrast through the ureteroarterial defect is documented along with subsequent opacification of the contiguous ureter. An alternative to arteriography is antegrade or retrograde ureterogram but it establish diagnosis in 52% and arteriography in 69%. CT can provide information but more often showing nonspecific findings such as blood clot formation in the renal pelvis and hydronephrosis rather than the discrete erosive structural wall defect necessary for diagnosis. Regarding treatments AUF requires multidisciplinary approach. Currently endovascular therapy is preferred to open surgery given the frequency of complicated anatomic presentation in most patients secondary to previous comorbidity [6].

On an urological level, once the vascular check has been carried out, the closure of the fistula orifice is most often treated with an endoureteral stent. However, it can be assumed that in this case that the ureter has not completely healed and the stent is in contact with infected urine, leading to thrombosis and the displacement of the stent and ultimately to the persistence of the source of infection, resulting in repeated pyelonephritis. Control uro-scanners did not give evidence of urinoma or renal abscess.

Upon withdrawal of the JJ catheter, we noted a significant decrease in renal excretion and the permeability of the ureter. Surgical principles for the treatment of fistulas recommend, as much as possible, the excision of the path and openings of the fistula and then interposing of a well-vascularized structure such as the omentum. The decision is based on the technical platform, the patient's medical history, and the degree of urgency. Endovascular treatment remains an emergency treatment and should, if possible, be followed up with open surgery because of the risk of thrombosis, fistula recurrence, and stent infection [1, 3].

In this case, the diagnosis of AUF was mentioned intraoperatively during for cystoscopy to massive hematuria with hemorrhagic shock. The placement of a balloon typically used for the treatment of stenoses of the ureter proved to be an effective solution to stop the bleeding. Given the number of surgical procedures performed previously on the abdomen and the hemodynamic status, surgical treatment was not considered this time.

To our knowledge, this technique has not been reported in this type of situation, but prolonged balloon use may cause ischemia or necrosis of the ureter. Remote infectious evolution suggests that the use of this technique should be limited to cases of absolute necessity. Stopping the bleeding during surgery was the main goal of treatment in order to allow the placement of a stent in optimal conditions. Our experience suggests that endovascular treatment must occur quickly after placement of the balloon. Later, questions remain regarding the efficiency of treatment on a urological level and the proper healing of the ureteral orifice.

## 4. Conclusion

Macroscopic hematuria in a patient bearing a long-term ureteral catheter and with a history of pelvic surgery and radiation therapy should suggest an FAU. In our case, the diagnosis was made intraoperatively during a bladder exploration with hemorrhagic shock. Bleeding was controlled by the introduction of a balloon catheter in the ureter and then by a vascular stent.
